# Welcoming and bonding in nursing care for homeless women in Primary Care

**DOI:** 10.1590/0034-7167-2023-0501

**Published:** 2025-10-03

**Authors:** Nayara Gonçalves Barbosa, Amanda Munhoz Garcia, Cinira Magali Fortuna, Tauani Zampieri Fermino, Lise Maria Carvalho Mendes, Thaís de Oliveira Gozzo, Lucila Castanheira Nascimento, Flávia Azevedo Gomes-Sponholz

**Affiliations:** I Universidade de São Paulo. São Paulo, São Paulo, Brazil; II Universidade de São Paulo. Ribeirão Preto, São Paulo, Brazil

**Keywords:** Women’s Health, Ill-Housed Persons, Primary Health Care, User Embracement, Nurse-Patient Relations., Salud de la Mujer, Personas con Mala Vivienda, Atención Primaria de Salud, Acogimiento, Relaciones Enfermero-Paciente.

## Abstract

**Objectives:**

to understand primary care nurses’ perceptions regarding the welcoming and building of bonds with homeless women.

**Methods:**

a descriptive, qualitative study, conducted with 17 nurses working in primary care in a large city in the countryside of the state of São Paulo. Semi-structured interviews were conducted, and the data were subjected to inductive thematic analysis.

**Results:**

three categories emerged: i) “*Weaving care: nurses’ support for homeless women”; ii) “Building bonds: support for comprehensive care”; iii) “Repercussions of caring for homeless women for nurses”*.

**Final Considerations:**

soft technologies provide support, bonding and comprehensive care for homeless women, guaranteeing the universal right to health and human dignity, fundamental elements for achieving the Sustainable Development Goals.

## RESUMEN

**Objetivos:**

comprender las percepciones de las enfermeras de atención primaria sobre la recepción y el desarrollo de vínculos con mujeres en situación de calle.

**Métodos:**

estudio descriptivo y cualitativo, realizado con 17 enfermeras de atención primaria en una gran ciudad del interior del estado de São Paulo. Se realizaron entrevistas semiestructuradas y los datos se sometieron a un análisis temático inductivo.

**Resultados:**

surgieron tres categorías: *i) “Tejiendo cuidados: el acompañamiento de enfermeras a mujeres en situación de calle”; ii) “Construyendo lazos: apoyo para la atención integral”; iii) “Repercusiones del cuidado a mujeres en situación de calle para enfermeras”.*

**Consideraciones Finales:**

las tecnologías blandas brindan apoyo, vínculo y atención integral a las mujeres en situación de calle, garantizando el derecho universal a la salud y la dignidad humana, elementos fundamentales para el logro de los Objetivos de Desarrollo Sostenible.

## INTRODUCTION

The condition of homeless people represents one of the most devastating examples of poverty, in a context of marginalization, social exclusion and expropriation of rights. According to the Brazilian National Policy for the Homeless Population, homeless people are those in a condition of extreme poverty, with the presence of interrupted or weakened family ties, lack of regular conventional housing and use of public roads and/or degraded areas as strategies for shelter or sustenance^(1)^.

Although Brazil does not officially count the homeless population, estimates indicate an increase of approximately 230% between 2012 and 2020, rising from 96,560 to 221,869, respectively^(2)^. Moreover, it is important to consider the worsening of this condition during the COVID-19 pandemic, which has accentuated social inequities and contributed to the increase in this population contingent^(3)^. The lack of systematic knowledge - quantitative and qualitative - about homeless people’s living conditions contributes to their invisibility and causes harm to the formulation of public policies^(4-6)^.

Although the homeless population is predominantly male^(3,4)^, women belong to one of the population groups in conditions of greatest vulnerability, in a context in which asymmetrical gender relations increase stigma and marginalization^(6)^. In this regard, they present complex health needs, considering the intersectionalities of gender, race and social class intertwined in the health-disease process^(7,8)^, and which play a structuring role in reproduction and production of social and subjective identity, relationships and social institutions^(8)^.

On the streets, women are subject to situations of violence, use of psychoactive substances, mental health problems, chronic degenerative conditions, exploitation, transactional sex, gynecological and reproductive problems, including a greater risk of acquiring sexually transmitted infections. They are also susceptible to unintended pregnancies and abortions in unsafe conditions, which favors the intergenerational perpetuation of poverty and misery^(9-12)^.

The defense of social rights and the right to health of homeless women is aligned with the Sustainable Development Goals (SDGs) regarding the issue of ensuring healthy lives, promoting well-being, eradicating poverty, and achieving gender equality, with a commitment to leaving no one behind^(13)^. In this way, it reinforces the need for the study to be carried out as a global priority.

Concerning care for the homeless population, the Brazilian National Primary Care Policy (In Portuguese, *Política Nacional de Atenção Básica* - PNAB) provides for the implementation of itinerant Street Clinic teams^(14)^. These teams deal directly with the different health problems and needs of the homeless population, through the development of on-site activities, on an itinerant basis, in dialogue with the Basic Health Units and other services that make up the healthcare network^(9,14)^. The PNAB also establishes the responsibility of homeless people in the territories covered by Basic Care teams, considering their specific needs and vulnerability conditions^(14)^.

These professionals work in a highly complex context and deal with political, economic and social issues^(15)^. This level of care is the gateway to the healthcare system and the locus of responsibility for providing care to users^(16)^, providing access to technologies capable of improving and prolonging life^(17)^.

The technologies that make up the work and production process of healthcare include the technologies inscribed in the instruments (hard technologies), structured technical knowledge (soft-hard technologies) and soft technologies, which refer to the relationships between subjects that only have materiality in action^(18,19)^. Soft technologies constitute practices of welcoming, listening and dialogue^(18-20)^, and represent a place of excellence in the care of populations in vulnerable situations, such as homeless women.

The results of this study are intended to encourage reflections on the relevance of soft and relational technologies in the comprehensive care provided by nurses to homeless women in the territories of Primary Care teams. The aim is to contribute to the recognition of soft technologies as essential tools to be used by nurses to guarantee access, acceptance, dignity, respect, defense of rights and attention to this population’s health needs.

## OBJECTIVES

To understand primary care nurses’ perceptions regarding the welcoming and building of bonds with homeless women.

## METHODS

### Ethical aspects

The study strictly followed the ethical precepts specified in Resolution 466 of December 12, 2012 of the Brazilian National Health Council for research conducted with human beings. The research was approved by a Research Ethics Committee linked to the Brazilian National Research Ethics Commission (In Portuguese, *Comissão Nacional de Ética em Pesquisa* - CONEP) on April 21, 2020. All participants signed the Informed Consent Form.

### Study design

This is a descriptive, exploratory and qualitative study. In order to comply with methodological rigor, the Consolidated Criteria for Reporting Qualitative Research (COREQ) guidelines were used^(21)^.

### Conceptual framework

This study is based on the concepts of welcoming and bonding as soft technologies of care. It is considered that health work is always relational, as it depends on live work in action, i.e., it occurs from the relationships established between working subjects and users, which occur during healthcare^(18,19)^. In this work, there is the presence of hard technologies, which are materials and equipment, soft-hard technologies, such as protocols and soft technologies, which are relational. Health work can be commanded by a type of technology predominantly and for producing comprehensive care it needs to be commanded by soft technologies, i.e., by attentive and interested listening, welcoming and bonding. Here, the concept of technology is understood by knowledge constituted for human organization in production processes^(18,19)^.

In health work, there is also the production of workers and users of healthcare services in the processes of production of subjectivities. There are encounters and also disagreements of different people with their different expectations, desires and needs, which requires a certain ethical and aesthetic positioning^(22)^.

Additionally, the study used precepts from the Brazilian National Policy for the Homeless Population^(1)^, the Brazilian National Primary Care Policy^(14)^ and the Brazilian National Humanization Policy^(23)^. From this perspective, when caring for the homeless population, it is essential to respect the dignity of human persons, the appreciation and respect for life and citizenship, humanized and universalized care, and above all, respect for social conditions and differences in origin, race, age, nationality, gender, sexual and religious orientation as well as for people with disabilities^(1)^.

The SDG guidelines also stand out for promoting health and well-being through humanized and universal care (SDG 3), gender equality (SDG 5), reducing inequalities by valuing the life and citizenship of all people, regardless of their personal characteristics (SDG 10), and respect for human dignity and citizenship, as they contribute to more just and inclusive societies (SDG 16). Strategic partnerships between the education, health and social assistance sectors, vital for the continuing training of professionals, ensure that they are able to face contemporary challenges with competency and sensitivity, fully aligning themselves with the objectives set by the SDGs (SDG 17)^(13)^.

### Study setting and participants

The study was conducted in four Basic Health Units, two Family Health Units and two School Health Centers in a large city located in the northwest region of the state of São Paulo. The health units were chosen because they were located in areas of greater social vulnerability and/or close to the central region, where a larger contingent of homeless people in the city is concentrated. Visits were made to the units during opening hours, and nurses were contacted to present the study objectives and invite them to participate in the research. Professionals who had experience in caring for homeless women in Primary Care services were included, and nurses who were on leave or vacation were excluded.

### Data collection, organization and analysis

Interviews were scheduled on a day and time convenient for participants. Individual interviews took place from July 2021 to February 2022, were audio-recorded, and lasted an average of 40 minutes. A single interview was conducted per participant, and all were conducted by an undergraduate nursing student, supervised by two PhDs in nursing with experience in qualitative research. The student had prior contact with Primary Care services through her participation in practical activities related to the undergraduate course subjects, but had no interaction or connection with the potential participants.

A semi-structured script was used, consisting of two parts: the first referred to participant characterization, such as age, sex, time since graduation and experience in Primary Care; the second part consisted of open-ended questions related to the care provided by nurses to homeless women in Primary Care. The questions were developed based on literature^(17-19)^ and the researchers’ experience, with the following guiding questions: *how do nurses and staff welcome homeless women in the region? How do they build bonds? What are the challenges in welcoming homeless women in Primary Care healthcare services and what are the potential opportunities?*

Participant recruitment interruption was supported by the sufficiency criterion, understood as the stage of research development in which the data begin to repeat themselves and this set of data allows us to respond to the study objective^(24)^. After data collection, the interviews were transcribed in full and the contents were ordered based on the transcription. To ensure anonymity, participants were identified by the letter N (nurse), followed by assigned Arabic numbers (from N1 to N17).

The data were subjected to inductive thematic analysis^(25)^. In the first stage of analysis, two researchers performed repeated readings of transcribed interviews to familiarize themselves with the data set. In the second stage, the initial codes were generated, from which the significant excerpts were highlighted in different colors, with the support of Microsoft Office Word^®^. In the third stage, the codes were grouped according to their affinities and relationships established to generate the initial topics. In the fourth phase, after refinement, descriptive topics were established and, subsequently, analytical topics were constructed, based on the synthesis of descriptive topics and articulations with the theoretical aspects of soft technologies. Senior researchers were consulted in case of disagreements. Finally, the final reflective analysis was carried out, based on the recognition of the understanding of the object of study and the articulation among topics with the literature and the researchers’ interpretation^(25)^.

## RESULTS

Seventeen nurses participated in the study, predominantly female (n=14; 82.35%), with time working in the service ranging from one month to eight years. Participants’ mean age was 42 years (±7.29), and the number of years of training in the undergraduate nursing course ranged from six to 13 years, with an average of 16 years (±6.77). All had specialization courses, and three (17.64%) had an academic master’s degree.

Three topics were constructed from the analytical process: i) “*Weaving care: nurses’ support for homeless women”;* ii) *“Building bonds: support for comprehensive care”;* iii) *“Repercussions of caring for homeless women for nurses”,* presented below:

### Weaving care: nurses’ support for homeless women

In the interviews, nurses pointed out aspects related to homeless women’s attitudes and elusive behavior as obstacles to approaching them and, therefore, to welcoming them.

*Homeless women are very aloof, some of them are very aggressive. Even when you go to talk to them, it’s a very complicated situation.* (N16)

*Sometimes,* [the woman] *is addicted to drugs, completely disturbed in relation to time, space and people, and she becomes more aggressive, without wanting to give any further explanations, and we need to listen to her at least in order to be able to resolve the issue.* (N8)

*Sometimes, we manage to build a bond with them, sometimes not. It depends, because homeless people themselves are resistant to the health team, and they don’t always welcome us well.* (N7)

Another aspect mentioned as interfering in this approach is the prejudice of some team members who receive homeless women at the health unit.

*Welcoming exists, the flow continues, there is the problem of the arrival of this type of population and the prejudices of those who are receiving these people.* (N17)

Also, in nurses’ statements, it is possible to identify that welcoming demands recognizing the singularities and vulnerability of these women.

*It is an extreme vulnerability, so I can’t just talk about the physical aspect, but also the psychological aspect.* [...] *there needs to be someone who listens, who welcomes, who provides emotional support so that this person can have adherence and self-care.* (N11)

*It is a special population, more vulnerable, we shouldn’t be prejudiced. We should treat them like normal citizens, like we treat everyone, but also looking at them in a special way.* (N9)

Nurses realize that approaching patients in an appropriate and private place and adapting language to allow women to better understand and identify with professionals are elements that favor interaction with these people.

*We try, as much as possible, because the physical space here is not helpful, but we try to talk more privately with these patients, otherwise they won’t open up.* (N1)

*I try to talk to her in a way that she herself talks, because if I start using jargon that she doesn’t understand it won’t work* [...]. *She has her own jargon, so I try to speak the same language as her.* (N16)

*First, we have to build a bond with her, sometimes the way we talk, even trying to use the slang that they use to see if we can make a connection. Then, after we manage to establish this bond, it becomes easier to work and for them to follow the instructions more.* (N12)

Welcoming is not restricted to the space “inside the walls” of Basic Health Units; it refers to professionals’ attitude of going to meet women in the territory, on the street, where they feel more comfortable expressing themselves.

*How can we build a bond? By welcoming someone. Where is this welcoming? Here in the classroom? No, sometimes we go and welcome them outside, on the street, because they don’t want to come in, we start talking and then bring them into the classroom. Or under the tree, on the bench, because they want to talk there. It has to be where they want, it’s not what we want.* (N2)

Welcoming homeless women involves identifying their complaints and recognizing them in their entirety and subjectivity. It means understanding that gestures, postures, looks and silences have meanings and can express health needs that must be translated and then met.

*Homeless women will not come to the welcoming, sometimes they will arrive here and will not even say that they are pregnant, or even introduce themselves. They will arrive wanting to go to the bathroom, to get a drink of water, they do not arrive looking for healthcare.* (N17)

### Building bonds: support for comprehensive care

Building bonds with these women occurs gradually, over time, through attentive listening, the exercise of non-judgment, cordial contact and acceptance, listening, human interactions and the construction of trusting relationships with the Primary Care team.

*Build this bond, have a connection with this woman to understand her needs and be able to know what she needs without her even saying it.* (N6)

*I usually ask her name, look her in the eyes, speak calmly and respect her time, then you can build a bond. But you have to go slowly, sideways.* (N3)

*You have to use conversational skills, it’s not really about pleasing her, but you have to make friends with her to be able to build a bond.* (N16)

Building bonds with homeless women is a challenge for nurses in Primary Care, since they do not have a fixed address, contacts or support network, which makes it difficult to continue providing care and actively seek out these women in the community. Therefore, it is necessary for them to recognize the Basic Health Unit as a reference and feel safe and confident to seek care on their own initiative.

*Regarding the challenges, I think it’s about building a bond so that she can seek out the unit, about building trust so that she can come without being discriminated against, because it’s going to be very difficult to find this woman, because she doesn’t have a fixed address, maybe she won’t have a phone number*. (N15)

*The biggest challenge is finding them, trying to build a bond with them, because we know that this is a population that gets lost every now and then, and there’s no contact.* (N9)

Building bonds is a sine qua non condition for continuity of care and helps women feel confident in attending and seeking out the health unit for attention to their needs.

*She has to find someone she can trust; if she trusts, she will come back. So, you have to keep your eyes open so you don’t let it go by, you can’t let it go by and think she will come back, because she won’t. So, you have to show her trust, because then maybe we can do something.* (N3)

*The thing is, sometimes, people who are homeless, drug users who are a bit altered, if their first impact with you was negative, they probably won’t come to you to have the first conversation; when they get to the unit, they will look for someone else who welcomed them the first time, you know? With more tact, with more skill.* (N12)

It is clear that, in addition to the health unit becoming a reference, professionals who welcome it also become a reference, as women always seek to be attended to by those who provide them with security and the possibility of building bonds.

*That professional who provides the first welcome, who starts talking, who treats her in a humane way, she will come to that professional. If she arrives at the unit, takes a look and sees that the professional is not there, she will leave. Because they have already encountered a lot of prejudice in the services, they report. So, when they arrive, they look for that professional with whom they have a connection.* (N2)

*When she arrives at the unit, she looks for the person she knows, who will help, who already knows the problems, with whom she has already built a connection.* (N4)

*Sometimes, they call us by name, sometimes we know them by name too, they are always the same.* (N1)

Professionals who build a bond are seen as a link with the team to promote comprehensive care for women.

*We try to identify the team member who has this connection, and we work on that professional. Because it is difficult to establish a connection with the team as a whole. They arrive and they are not open to this type of connection. But it is always some professional who manages this type of thing, and this professional ends up being the link. Not necessarily nurses, sometimes it is the CHWs, the nursing assistants who established the connection, and this will be the bridge.* (N7)

*It is important to have a connection with some team member, to build this connection with this woman so that she can be a reference within the unit. It can even be the general services, from the cleaning lady to the doctor so that she can have this trust and feel welcomed.* (N6)

The potential of the bond has significant impacts on women’s care, promoting joint team efforts in mobilizing strategies to seek solutions and meet health needs.

*I can build a bond. It has happened several times that we have this bond and are able to be efficient in care, in resolving the problem and there have even been happy cases where we have managed to get the woman out of that situation of drug addiction, out of the streets and she has found a job.* (N6)

### Repercussions of caring for homeless women for nurses

Caring for homeless women can sensitize nurses and cause emotional repercussions.

*Today I understand that* [...] *so as not to take on the problem myself. Because this really makes our work as nurses more difficult. Even because we women identify with the issue of being female, so it really ends up hurting a little.* (N6)

*It’s a little disconcerting, because you see that it’s a human being, most of the time they are women of a young, fertile age and already in a situation of being lost in some aspects of their lives.* (N8)

*I think the whole team is moved. It’s impossible not to be moved.* (N10)

The care provided by nurses to homeless women promotes reflections on quality of care, in order to identify what could be done beyond what is already done for this population, in order to meet their health needs and promote comprehensive care.

*I feel like, “Oh my God, what more could I do?”. It feels like something is missing. What more could I do to help? I feel like there is a lack of work with homeless women, in particular.* (N13)

*It is impossible not to be sensitive and not to think about what you can do differently.* (N12)

*Sometimes, I feel devastated, I feel bad. I attended to one* [user] *and I didn’t know she was homeless and I didn’t have a very welcoming look at her at that moment, so much was happening at this health center* [Basic Health Unit]. *After* [nursing consultation] *was over, they told me that she was homeless and that the main problem was that she didn’t have food. That day I left here devastated. I said, “People, how did I not see this?”.* (N3)

In this regard, nurses recognize the need for in-service training and initial training of professionals to welcome homeless people and identify specificities in the care of women.

*My main difficulty is knowing how to talk to them, what to ask them. I think there is a barrier. They are already a bit insecure and I end up feeling the same way about how to approach them. I don’t know the best way to approach them and be able to talk better, really get to know the person, gain their trust. I confess that I myself would need training, qualification for this.* (N12)

*I think more training is needed, because in college itself, they don’t teach how to welcome a homeless person, especially because the service is very complex.* (N2)

## DISCUSSION

The conditions of vulnerability concerning homeless women are expressed in multiple health needs^(17)^, with the lack of good living conditions, inaccessibility to appropriate technologies for managing suffering, fragility in relationships of affection and bonds with the health team and limited autonomy to lead one’s life. Hence, it becomes essential to strengthen the use of soft technologies as a therapeutic instrument in the care of these women, whose humanity and social rights are violated on a daily basis^(12)^. Promoting health and well-being for all, especially for vulnerable populations, is a key objective of the SDGs. The use of soft technologies as therapeutic tools is aligned with the goal of improving homeless women’s mental health and well-being as well as respecting human dignity and promoting citizenship rights^(13)^.

The streets are, among so many existential territories, a place where existences act and are produced as living networks^(26)^. The streets present specific and plural codes, they contain joys, pains, disappointments, challenges and tensions that constitute life and death production in the daily life of walking through life^(6,26)^. They are expressed in a “street culture”, with singularities and their own conformations, which are usually strange to health workers, and they challenge institutional knowledge and the daily practices of healthcare services when dealing with their “unusual” existences, to build a proposal for common care, i.e., shared between workers and users^(6)^.

Encounters between health workers and homeless people can occur in a context permeated by tension, aggression and distrust^(5,12,27,28)^. Often, due to the inhospitable conditions experienced by these women on the streets, as well as negative experiences in healthcare services, trauma and the effects of substances, they exhibit protective, aggressive behaviors and a tendency to keep their distance from people^(12,28)^. In this sense, nurses and the health team may encounter resistance to approaching each other and, constantly, based on constitutive tensions, negotiations take place between users and services^(26)^.

Studies point to reports of prejudice, discrimination and social devaluation of homeless people by healthcare professionals^(5,27)^. Research also reveals that homeless women have a history of having suffered violence and that, in their daily lives on the streets, they continue to suffer violence, perpetrated, including by the institutions that should protect them, such as the police^(29)^. In addition, some sanitary actions of a hygienic and punitive nature are present in public spaces^(30)^.

The resistance cited by nurses by homeless women and health teams can be interpreted as the result of a cycle of violence and discrimination that needs to be broken, and it is expected that healthcare professionals will take the initiative to do so. Listening with a non-judgmental approach can help, but prejudice and resistance in caring for homeless people must be recognized^(26)^.

Welcoming was a soft technology recognized by the interviewees for developing a bond with homeless women, which, in a context of first contact and longitudinality to healthcare services, contributes to the reduction of inequalities^(15)^, a *sine qua non* condition for achieving the SDGs. In this case, attention to homeless women’s spontaneous demands, such as gynecological complaints, represents an opportunity for comprehensive care, as it allows the identification and attention to other health needs from a broader and deeper perspective^(9)^. Thus, welcoming favors nurses’ clinical practice, the development of health-promoting actions, teamwork, interprofessional and user relationships, and improved access^(15)^. The proposal operationalization requires guidance from teams so that they take responsibility of welcoming users in their demands, listening, negotiating and agreeing on care projects^(20)^.

Many homeless women do not remain in a defined geographic area, which calls on healthcare professionals to be more open about the rules for defining their target clientele, and for requiring documents and proof of address. The complexity of this population’s health needs demands care technologies that transcend the usual routines and protocolized conduct, guided by a bureaucratic organizational logic that tends to standardize the provision of care^(6,26)^. This logic also does not ensure that homeless women’s specificities and singularities are met, and may even constitute a barrier to access to healthcare services^(27)^.

The concept of existential territory can be a guiding axis for the production of bonds between nurses and healthcare professionals in the production of care for women living on the streets. The valorization of the territory for the meeting of interlocutors, with space for listening and speaking, with the potential to welcome different understandings of ways of the world, provides the opening of new paths for interference and interventions, repercussing on all the subjects involved as well as on the process of knowledge production^(6)^.

This indicates the presence of “unusual” modes of existence produced by users, which are often judged and restricted by health teams. Consequently, they are trapped in a way of knowing that is so predominant that it limits the recognition that certain attitudes, behaviors and expressions are also modes of existence, even if they are full of tensions and problems^(26)^. There is evidence of frustration among health workers and teams, as well as the feeling of failure when carrying out actions in adverse scenarios, which can precipitate abandonment or the taking of purely prescriptive decisions and conduct, reducing the possibilities of establishing a bond^(6)^.

Furthermore, as a limiting factor, the welfare culture’s influence stands out, whose scope of government actions is limited to immediate problems: shelter, food and clothing^(6)^. Working towards the perspective of guaranteeing social rights remains a challenge for governments and their care networks^(6)^. The implementation of dignified and respectful care for homeless people does not refer to altruism, compassion or professional kindness; it is a matter of user rights, guaranteed by the Federal Constitution and the SUS^(1,14,23)^.

One of the challenges faced by Primary Care nurses in caring for homeless women is precisely building bonds. This is considered a soft technology, which refers to a close and lasting relationship between professionals and users, with emotional ties that allow for continuity of care^(16)^. Building (a)effective bonds between each user and the team and/or health worker^(17)^ is essential to address homeless women’s health needs.

The way of operating health work, from a perspective of symmetry in the encounter between different people, values the unique potential of each individual as a fundamental material for the production of care^(6)^. In encounters permeated by intersubjectivities, it becomes essential to know women and address them by their preferred name and/or nickname, since living on the streets transforms subjects’ personal and social identity, promoting their invisibility, exclusion and low self-esteem^(12)^. From this perspective, valuing women’s identity refers to the encounter and recovery of their human essence, dignity and citizenship^(12,28)^.

Care also involves giving these women a voice, valuing them, knowing their life stories, the reasons for finding themselves homeless, the ways they (sur)vive and navigate life on the streets, as well as understanding their social and family relationships, their singularities and life plans^(12,28)^. It is important to note that users, as networks of existences, are produced “in worlds”, “in-worlding”, constituting distinct existential ethical expressions and ways of conducting, by themselves, care production^(26)^. Incorporating the design of the multiplicity of lives into projects for approaching and caring for women living on the streets is a technology/knowledge/practice to be incorporated by professionals in social policies^(6)^.

The appreciation of these encounters, in depth, allows for mutual affect, affecting and being affected, activating strategies for living that bet on the power of life, based on the joint construction of solutions to problems, on a common path for both parties^(6)^. The ethical and aesthetic premise is that every form of life has the right to express itself fully and it is up to healthcare services to provide access and support, especially to these women who certainly express social, gender, racial and other inequality in intersectionality. In this direction, soft technologies present themselves as practices of welcoming, listening and dialogue, in a privileged field for exercising self-government and bringing healthcare closer to people’s life projects^(20)^. Ensuring that all life has the right to express itself to its fullest is related to the promotion of peaceful and inclusive societies, as set out in SDG 16. Effective and fair health institutions are fundamental to ensuring respect for human rights^(13)^.

In this sense, it is beneficial to develop strategies for continuing education in health to problematize work processes, analyzing the welcoming of homeless women. The interviewees’ speeches identified the recognition of the need for training and improvement for care. From this perspective, the importance of nurses’ citizenship training stands out, considering their social role, with a critical and reflective view, aware of their technical, scientific, ethical and political performance^(31)^ in the care of homeless women. The joint construction of solutions and the importance of continuing training for healthcare professionals highlight the need for effective partnerships with educational institutions and non-governmental organizations. This is a collective effort to transform care and social inclusion, in line with SDG 17^(13)^, in order to promote inclusive and equitable public health, which is fundamental for sustainable development and social justice. This requires coordinated and multisectoral action to address the complex realities experienced by homeless women.

Nursing professionals are key players in transforming global goals into tangible actions and in promoting a healthier, more equitable and sustainable world. In this way, nursing professionals in primary care services can develop professional practices aligned with and committed to the SDGs in their daily lives, acting as an essential pillar in promoting health and combating inequalities. They can also play a vital role in promoting health, gender equality, reducing inequalities and building more just and peaceful societies by ensuring equal access to quality healthcare.

### Study limitations

The present study had limitations because it was restricted to interviews with nurses, without observing the care provided.

### Contributions to the area

This study encourages reflections on the relevance of using soft technologies in the care of homeless women in primary healthcare, considering the complexity of their health needs and how living conditions on the streets impact this population’s health-disease process and biopsychosocial well-being. Nursing training at the undergraduate and graduate levels, in addition to in-service training, is essential to provide support for the welcoming and comprehensive care of an invisible and marginalized population, such as homeless women.

## FINAL CONSIDERATIONS

The use of soft care technologies in Primary Care nurses’ daily work, with the use of relational tools in healthcare, has the potential to foster the building of bonds of trust and dialogue between homeless women and nurses and teams. It means expanding care beyond the space “inside the walls” of Basic Health Units and meeting women in their territory, on the streets, recognizing their rights and needs. It is considered that health work, as a relational work, depends on live work in action, i.e., it occurs from the relationships established between workers and users, which occur during healthcare. Soft technologies can command health work through the production of bonds and welcoming, a fundamental aspect for the recognition of the rights of women living on the streets and in vulnerable situations. Furthermore, the importance of in-service training and initial training of professionals for the development of relational skills, the recognition of welcoming and connection as essential technologies for the care of women living on the streets stands out. These elements are fundamental for the welcoming and comprehensive care of women living on the streets and for guaranteeing the right to health and human dignity, fundamental elements for the construction of a better world, a more just and inclusive society and for the achievement of the SDGs.

## Data Availability

The research data are available within the article.
