# Supplementary material for: Welcoming and bonding in nursing care for homeless women in Primary Care
Source: Rev Bras Enferm. 2025 Oct 3;78(Suppl 2):e20230501. doi: 10.1590/0034-7167-2023-0501 (PMC12500075; doi:10.1590/0034-7167-2023-0501)
Supplement: Translation

# Translation: Acolhimento e vínculo no cuidado do(a) enfermeiro(a) a mulheres em situação de rua na Atenção Básica

**DOI:** 10.1590/0034-7167-2023-0501pt

**Authors:** Nayara Gonçalves Barbosa, Amanda Munhoz Garcia, Cinira Magali Fortuna, Tauani Zampieri Fermino, Lise Maria Carvalho Mendes, Thaís de Oliveira Gozzo, Lucila Castanheira Nascimento, Flávia Azevedo Gomes-Sponholz

**Affiliations:** I Universidade de São Paulo. São Paulo, São Paulo, Brasil; II Universidade de São Paulo. Ribeirão Preto, São Paulo, Brasil

**Keywords:** Saúde da Mulher, População em Situação de Rua, Atenção Primária à, Saúde, Acolhimento, Relações Enfermeiro-Paciente.

## Abstract

**Objetivos:**

conhecer as percepções de enfermeiros da Atenção Básica quanto ao acolhimento e à construção de vínculo com mulheres em situação de rua.

**Métodos:**

estudo descritivo, qualitativo, realizado com 17 enfermeiros(as) atuantes na Atenção Básica de um município de grande porte do interior do estado de São Paulo. Foram realizadas entrevistas semiestruturadas, e os dados submetidos à análise temática indutiva.

**Resultados:**

emergiram três categorias: i) “*Tecendo o cuidado: o acolhimento dos enfermeiros a mulheres em situação de rua”;* ii) *“Construção de vínculo: subsídio para a integralidade do cuidado”;* iii) *“Repercussões do cuidado de mulheres em situação de rua para os enfermeiros”*.

**Considerações Finais:**

as tecnologias leves propiciam acolhimento, criação de vínculos e cuidado integral a mulheres em situação de rua, na garantia do direito universal à saúde e da dignidade humana, elementos fundamentais para o alcance dos Objetivos do Desenvolvimento Sustentável.

## INTRODUÇÃO

A condição das pessoas em situação de rua representa um dos exemplos mais devastadores da pobreza, em um contexto de marginalização, exclusão social e expropriação de direitos. De acordo com a Política Nacional para a População em Situação de Rua, denominam-se pessoas em situação de rua aquelas em condição de extrema pobreza, com presença de vínculos familiares interrompidos ou fragilizados, ausência de moradia convencional regular e utilização de vias públicas e/ou áreas degradadas como estratégias para abrigo ou sustento^(1)^.

Embora o Brasil não realize contagem oficial da população em situação de rua, estimativas apontam um aumento de aproximadamente 230% entre 2012 e 2020, subindo de 96.560 para 221.869, respectivamente^(2)^. Além disso, deve-se considerar o agravamento desta condição durante a pandemia de COVID-19, que acentuou as iniquidades sociais e contribuiu para o aumento desse contingente populacional^(3)^. A escassez de conhecimento sistematizado - quantitativo e qualitativo - sobre as condições de vida das pessoas em situação de rua corrobora para a sua invisibilidade e acarreta prejuízos na formulação de políticas públicas^(4-6)^.

Apesar da população em situação de rua ser majoritariamente masculina^(3,4)^, as mulheres pertencem a um dos grupos populacionais em condições de maior vulnerabilidade, em um contexto no qual as relações assimétricas de gênero potencializam o estigma e a marginalização^(6)^. Nessa direção, elas apresentam necessidades de saúde complexas, considerando as interseccionalidades de gênero, raça e classe social imbricadas no processo saúde-doença^(7,8)^, que exercem papel estruturante na reprodução e produção da identidade social e subjetiva, das relações e das instituições sociais^(8)^.

Nas ruas, as mulheres estão sujeitas a situações de violência, uso de substâncias psicoativas, agravos de saúde mental, condições crônico-degenerativas, exploração, prática de sexo transacional, problemas ginecológicos e reprodutivos, incluindo maior risco de aquisição de infecções sexualmente transmissíveis. Destaca-se ainda a suscetibilidade à ocorrência de gestações não intencionais e de abortamento em condições inseguras como fator que favorece a perpetuação intergeracional da pobreza e da miséria^(9-12)^.

A defesa dos direitos sociais e do direito à saúde das mulheres em situação de rua encontra-se alinhada com os Objetivos de Desenvolvimento Sustentável (ODS) no que tange a aspectos como garantia de uma vida saudável, promoção da saúde e bem-estar, erradicação da pobreza e igualdade de gênero, sem excluir qualquer pessoa^(13)^. Dessa forma, fortalece-se a necessidade de realização do estudo enquanto prioridade global.

No que se refere à atenção à população em situação de rua, a Política Nacional de Atenção Básica (PNAB) prevê a implementação de equipes itinerantes de Consultórios na Rua^(14)^. Essas equipes lidam diretamente com os diferentes problemas e necessidades de saúde da população em situação de rua, a partir do desenvolvimento de atividades *in loco*, de forma itinerante, em interlocução com as Unidades Básicas de Saúde e demais serviços que compõem a rede de atenção à saúde^(9,14)^. A PNAB estabelece ainda a responsabilização das pessoas em situação de rua nos territórios adscritos de atuação das equipes de Atenção Básica, considerando suas necessidades específicas e condições de vulnerabilidades^(14)^.

Esses profissionais atuam em um contexto de alta complexidade e lidam com questões políticas, econômicas e sociais^(15)^. Este nível de atenção configura-se como a porta de entrada ao sistema de saúde e *lócus* de responsabilidade pela atenção aos usuários^(16)^, propiciando acesso a tecnologias capazes de melhorar e prolongar a vida^(17)^.

As tecnologias que compõem o processo de trabalho e de produção do cuidado em saúde incluem aquelas inscritas nos instrumentos (tecnologias duras), o saber técnico estruturado (tecnologias leve-duras) e as tecnologias leves, que se referem às relações entre sujeitos que só têm materialidade em ato^(18,19)^. As tecnologias leves constituem as práticas de acolhimento, escuta e diálogo^(18-20)^ e representam um lugar de excelência no cuidado de populações em situação de vulnerabilidade, como é o caso de mulheres em situação de rua.

Intenciona-se, com os resultados deste estudo, fomentar reflexões acerca da relevância das tecnologias leves e relacionais no cuidado integral do(a) enfermeiro(a) a mulheres em situação de rua nos territórios das equipes de Atenção Básica. O intuito é contribuir para que as tecnologias leves sejam reconhecidas como ferramentas essenciais a serem utilizadas por enfermeiros(as) para garantir o acesso, acolhimento, dignidade, respeito, defesa de direitos e atenção às necessidades de saúde dessa população.

## OBJETIVOS

Conhecer as percepções de enfermeiros(as) da atenção básica quanto ao acolhimento e à construção de vínculo com mulheres em situação de rua.

## MÉTODOS

### Aspectos éticos

O estudo seguiu rigorosamente os preceitos éticos especificados na Resolução nº 466, de 12 de dezembro de 2012, do Conselho Nacional de Saúde para pesquisas realizadas com seres humanos. Foi aprovado por um Comitê de Ética em Pesquisa vinculado à Comissão Nacional de Ética em Pesquisa (CONEP) em 21 de abril de 2020. A assinatura ao Consentimento Livre e Esclarecido foi obtida de todos os participantes do estudo.

### Tipo de estudo

Estudo descritivo, exploratório, de abordagem qualitativa. Para o cumprimento do rigor metodológico, foram utilizadas as diretrizes do Critério Consolidado de Relato de Pesquisa Qualitativa (COREQ)^(21)^.

### Quadro conceitual

Este estudo se fundamenta nos conceitos de acolhimento e vínculo enquanto tecnologias leves do cuidado. Considera-se que o trabalho na saúde é sempre relacional, pois depende do trabalho vivo em ato, ou seja, ocorre a partir das relações estabelecidas entre sujeitos trabalhadores e usuários, que acontecem durante o cuidado à saúde^(18,19)^. Nesse trabalho, estão presentes tecnologias do tipo dura, que são materiais e equipamentos, tecnologias leve-duras, como os protocolos, e tecnologias leves, que são aquelas relacionais. O trabalho em saúde pode ser comandado por um tipo de tecnologia predominantemente e, para a produção do cuidado integral, precisa ser comandado pelas tecnologias leves, ou seja, pela escuta atenta e interessada, pelo acolhimento e pelo vínculo. Aqui, o conceito de tecnologia é compreendido por saberes constituídos para a organização humana nos processos produtivos^(18,19)^.

No trabalho em saúde, ocorre também a produção dos trabalhadores e usuários dos serviços de saúde nos processos de produção de subjetividades. São encontros e também desencontros de diferentes pessoas com suas distintas expectativas, desejos e necessidades, o que requer um certo posicionamento tanto ético como estético^(22)^.

Adicionalmente, o estudo utilizou preceitos da Política Nacional para a População em Situação de Rua^(1)^, da Política Nacional de Atenção Básica^(14)^ e da Política Nacional de Humanização^(23)^. Nessa perspectiva, no cuidado à população em situação de rua, torna-se imprescindível manter o respeito à dignidade da pessoa humana, a valorização e respeito à vida e à cidadania, atendimento humanizado e universalizado e, sobretudo, o respeito às condições sociais e diferenças de origem, raça, idade, nacionalidade, gênero, orientação sexual e religiosa, bem como às pessoas com deficiência^(1)^.

Destacam-se também as orientações dos ODS para promoção da saúde e do bem-estar, por meio de um atendimento humanizado e universalizado (ODS 3), igualdade de gênero (ODS 5), redução das desigualdades, ao valorizar a vida e a cidadania de todas as pessoas, independentemente de suas características pessoais (ODS 10), e respeito à dignidade humana e à cidadania, pois contribuem para sociedades mais justas e inclusivas (ODS 16). As parcerias estratégicas entre os setores da educação, saúde e assistência social, vitais para a formação contínua dos profissionais, garantem que estes estejam aptos a enfrentar os desafios contemporâneos com competência e sensibilidade, alinhando-se plenamente aos objetivos traçados pelos ODS (ODS 17)^(13)^.

### Cenário e participantes do estudo

O estudo foi desenvolvido em quatro Unidades Básicas de Saúde, duas Unidades de Saúde da Família e dois Centros de Saúde Escola de um município de grande porte localizado na região noroeste do estado de São Paulo. Essas unidades foram escolhidas em detrimento de suas localizações em áreas de maior vulnerabilidade social e/ou próximas à região central, na qual se concentram maior contingente de pessoas em situação de rua no município. Foram realizadas visitas nas referidas unidades em horário de funcionamento, além de contato com os(as) enfermeiros(as) para apresentar os objetivos do estudo e convidá-los(as) a participar da pesquisa. Foram incluídos(as) profissionais com experiência no atendimento a mulheres em situação de rua nos serviços de Atenção Básica e excluídos(as) enfermeiros(as) no período de afastamento ou de férias.

### Coleta, organização e análise dos dados

Procedeu-se ao agendamento das entrevistas em dia e horário convenientes aos participantes. As entrevistas individuais ocorreram de julho de 2021 a fevereiro de 2022, foram audiogravadas e tiveram duração média de 40 minutos. Foi realizada uma única entrevista por participante, e todas foram conduzidas por uma aluna de graduação do curso de bacharelado em enfermagem, supervisionada por duas doutoras em enfermagem, com experiência em pesquisa qualitativa. A estudante possuía contato prévio com os serviços de Atenção Básica por meio de sua inserção em atividades práticas vinculadas às disciplinas do curso de graduação, mas não tinha interação ou vínculo com os potenciais participantes.

Utilizou-se um roteiro semiestruturado composto por duas partes: a primeira referente à caracterização dos participantes, tais como idade, sexo, tempo de formação e atuação na Atenção Básica, e a segunda com questões abertas relacionadas aos cuidados do enfermeiro a mulheres em situação de rua na Atenção Básica. As questões foram elaboradas com base na literatura^(17-19)^ e na experiência das pesquisadoras, com as seguintes questões norteadoras: *Como é o acolhimento do enfermeiro e da equipe a mulheres em situação de rua no território? Como é a criação de vínculos? Quais são os desafios no acolhimento à mulher em situação de rua nos serviços de saúde no âmbito da Atenção Básica e quais as potencialidades?*

A interrupção do recrutamento dos participantes foi subsidiada pelo critério de suficiência, entendido como a etapa do desenvolvimento da pesquisa em que os dados começam a se repetir e esse conjunto de dados permite responder ao objetivo do estudo^(24)^. Após a coleta dos dados, realizou-se a transcrição integral das entrevistas, e os conteúdos foram ordenados a partir da transcrição. Para garantia do anonimato, os participantes foram identificados pela letra E (enfermeiro/a), seguida por números arábicos atribuídos conforme a ordem de realização das entrevistas (de E1 a E17).

Os dados foram submetidos à análise temática indutiva^(25)^. Na primeira etapa da análise, duas pesquisadoras realizaram leituras repetidas das entrevistas transcritas para a familiarização com o conjunto de dados. Na segunda etapa, foram gerados os códigos iniciais, a partir dos quais os extratos significativos foram destacados com cores diferentes, com o apoio da ferramenta do *Microsoft Office Word* ^®^. Na terceira fase, procedeu-se ao agrupamento dos códigos, segundo as suas afinidades e relações estabelecidas para a geração dos temas iniciais. Na quarta fase, após refinamento, foram estabelecidos os temas descritivos e, subsequentemente, procedeu-se à construção dos temas analíticos, a partir da síntese dos temas descritivos e articulações com os aspectos teóricos das tecnologias leves. Pesquisadores seniores foram consultados em caso de divergências. Por fim, realizou-se a análise reflexiva final, a partir do reconhecimento da compreensão do objeto de estudo e da articulação entre os temas com a literatura e a interpretação das pesquisadoras^(25)^.

## RESULTADOS

Participaram do estudo 17 enfermeiros(as), predominantemente do sexo feminino (n=14; 82,35%), com tempo de atuação no serviço variando de um mês a oito anos. A média de idade dos participantes foi de 42 anos (±7,29), e a de formação no curso de graduação em Enfermagem variou de seis anos a 13 anos, com média de 16 anos (±6,77). Todos possuíam cursos de especialização e três (17,64%) mestrado acadêmico.

A partir do processo analítico, foram construídos três temas: i) “*Tecendo o cuidado: o acolhimento dos enfermeiros a mulheres em situação de rua”;* ii) *“Construção de vínculo: subsídio para a integralidade do cuidado”;* iii) *“Repercussões do cuidado de mulheres em situação de rua para os enfermeiros”,* apresentados a seguir:

### Tecendo o cuidado: o acolhimento dos enfermeiros a mulheres em situação de rua

Nas entrevistas, os(as) enfermeiros(as) apontaram aspectos relacionados às atitudes e ao comportamento esquivo das mulheres em situação de rua como obstáculos para uma aproximação e, portanto, para o acolhimento.

*As mulheres em situação de rua são bem arredias, tem algumas que são bem agressivas. Até na hora que você vai conversar com elas é uma situação bem complicada.* (E16)

*Às vezes,* [a mulher] *está em situação de drogadição, totalmente transtornada com relação a tempo, espaço e pessoa, já vem mais agressiva, sem querer dar maiores explicações, e a gente precisa do mínimo da escuta pra poder dar uma resolutividade.* (E8)

*Às vezes, a gente consegue criar um vínculo com elas, às vezes não. Depende, porque as pessoas em situação de rua em si têm uma resistência com a equipe de saúde, nem sempre eles nos recebem bem.* (E7)

Outro aspecto mencionado como interferente nessa aproximação é o preconceito de alguns membros da equipe que recebem as mulheres em situação de rua na unidade de saúde.

*O acolhimento existe, o fluxo segue, existe o problema da chegada desse tipo de população e os preconceitos de quem está recebendo essas pessoas.* (E17)

Também, nas falas dos(as) enfermeiros(as), identifica-se que o acolhimento demanda reconhecer as singularidades e vulnerabilidade dessas mulheres.

*É uma extrema vulnerabilidade, então não posso falar só do físico, mas do psicológico também.* [...] *Falta um alguém que ouça, que acolha, ter um suporte emocional pra essa pessoa conseguir ter uma adesão e autocuidado.* (E11)

*É uma população especial, mais vulnerável, a gente não tem que ter preconceito. Tem que tratar ele como um cidadão normal, como a gente trata a todos, mas olhando também de uma maneira especial pra eles.* (E9)

Os(as) enfermeiros(as) percebem que a abordagem em um local apropriado e reservado e a adaptação da linguagem para melhor compreensão e identificação da mulher com o profissional são elementos que favorecem a interação com essas pessoas.

*A gente tenta, na medida do possível, porque aqui o espaço físico não ajuda, mas a gente tenta falar de forma mais reservada com essas pacientes, se não elas não se abrem.* (E1)

*Eu tento conversar com ela de uma maneira como ela mesma conversa, porque se eu começar a usar um palavreado que ela não entende não vai resolver* [...]*. Ela tem o palavreado próprio dela, então eu tento falar a mesma língua que ela.* (E16)

*Primeiro a gente tem que criar um vínculo com ela, às vezes a maneira de conversar, até tentar usar as gírias que elas usam pra ver se a gente faz uma conexão. Aí, depois que a gente consegue estabelecer esse vínculo, fica mais fácil de trabalhar e delas seguirem mais as orientações.* (E12)

O acolhimento não se restringe ao espaço “intramuro” da unidade básica de saúde; refere-se à postura do profissional, de ir ao encontro da mulher no território, na rua, onde ela se sente mais à vontade para expressar-se.

*Como que a gente consegue construir vínculo? Com acolhimento. Onde esse acolhimento? Aqui na sala? Não, às vezes a gente vai acolher lá fora mesmo, na rua, porque ela não quer entrar, começa a conversar pra depois trazer ela pra dentro da sala. Ou embaixo da árvore, lá no banco, porque ela quer conversar lá. Tem que ser onde ela quer, não é o que nós queremos.* (*E2*)

O acolhimento à mulher em situação de rua perpassa a identificação das queixas, com a necessidade de reconhecê-la em sua integralidade e subjetividades. Significa compreender que os gestos, posturas, olhares, silêncios possuem significados e podem expressar necessidades de saúde a serem traduzidas e, em seguida, atendidas.

*A mulher em situação de rua não vai chegar até a recepção, às vezes vai chegar aqui e nem vai falar que está grávida, nem se apresenta, ela vai chegar querendo ir no banheiro, ir tomar uma água, ela não chega procurando assistência à saúde.* (E17)

### Construção de vínculo: subsídio para a integralidade do cuidado

A criação de vínculos com essas mulheres ocorre paulatinamente, ao longo do tempo, a partir da escuta atenta, do exercício do não julgamento, do contato cordial e acolhimento, da escuta, das interações humanas e da construção de relações de confiança com a equipe de Atenção Básica.

*Criar esse vínculo, ter um vínculo com essa mulher, pra entender essas necessidades e conseguir saber o que ela precisa sem ao menos ela falar.* (E6)

*Eu costumo perguntar o nome delas, olhar nos olhos delas, falar com calma e respeitar o tempo delas, daí você consegue criar um vínculo sim. Só que você tem que ir devagar, pelos ladinhos.* (E3)

*Você tem que usar o manejo da conversa, não é bem um agradar, mas você tem que ali fazer uma amizade com elas pra conseguir criar um vínculo.* (E16)

A criação de vínculos com as mulheres em situação de rua representa um desafio para o(a) enfermeiro(a) na Atenção Básica, uma vez que não possuem endereço fixo, contatos ou rede de apoio, o que dificulta a continuidade do cuidado e a busca ativa dessas mulheres na comunidade. Assim, é necessário que reconheçam a Unidade Básica de Saúde como uma referência e sintam segurança e confiança para buscar o cuidado, por iniciativa própria.

*Sobre os desafios, eu acho que é a gente criar o vínculo para ela procurar a unidade, da gente criar confiança dela poder vir sem ser discriminada, porque vai ser muito difícil encontrarmos essa mulher, por ela não ter um endereço fixo, talvez não vai ter nenhum telefone.* (E15)

*A maior dificuldade mesmo é a localização delas, tentar criar um pacto com elas de vínculo mesmo, porque a gente sabe que é uma população que vira e mexe a gente se perde, não tem um contato.* (E9)

A construção dos vínculos é condição *sine qua non* para a continuidade do cuidado e contribui para que a mulher sinta confiança em frequentar e ir até a unidade de saúde para atenção às suas necessidades.

*Ela tem que conseguir alguém em quem ela possa ter confiança, se ela tem confiança ela volta. Então você tem que abrir seu olho pra não deixar passar, não dá pra deixar passar e pensar que ela vai voltar, porque não volta. Então você tem que demonstrar confiança pra ela, porque daí quem sabe a gente consegue fazer alguma coisa.* (E3)

*É que, às vezes, a pessoa em situação de rua de rua, usuária de drogas que tá meio alterada, se o primeiro impacto com você já foi negativo, provavelmente ela não vai mais buscar você pra ter a primeira conversa, quando ela chegar na unidade ela vai buscar outra pessoa que acolheu ela de primeira, sabe? Com mais tato, com mais jeito.* (E12)

Denota-se que, além da unidade de saúde tornar-se uma referência, o profissional que a acolhe também se torna uma referência, pois a mulher sempre busca ser atendida por quem que lhe forneceu segurança e possibilidade de construção de vínculo.

*Aquele profissional que faz o primeiro acolhimento, que começa a conversar, que trata ela de uma forma humana, ela vai vir naquele profissional. Se ela chegar na unidade, der uma olhada e ver que o profissional não tá, ela vai embora. Porque elas já se depararam muito com muito preconceito nos serviços, elas relatam. Então, quando elas chegam, elas procuram aquele profissional que ela tem vínculo.* (E2)

*Quando ela chega na unidade ela já procura a pessoa que ela sabe que conhece, que vai ajudar, que já sabe os problemas, que já criou um vínculo.* (E4)

*Às vezes, nos chamam pelo nome, às vezes a gente conhece elas pelo nome também, são sempre as mesmas.* (E1)

O profissional que constrói o vínculo é tido como um elo com a equipe, para promover o cuidado integral para a mulher.

*A gente tenta detectar aquele membro da equipe que teve esse vínculo, e trabalha em cima daquele profissional. Porque, dificilmente, faz vínculo com a equipe como um todo. Elas chegam e elas não são abertas a esse tipo de vínculo. Mas é sempre algum profissional que consegue esse tipo de coisa e esse profissional acaba sendo o elo. Não necessariamente o enfermeiro, às vezes é o ACS, o auxiliar de enfermagem que fez o vínculo, e essa vai ser a ponte.* (E7)

*É importante ter o vínculo com algum membro da equipe, criar esse vínculo com essa mulher, pra que seja uma referência dentro da unidade. Pode ser até mesmo a serviços gerais, desde a faxineira até o médico, pra ela ter essa confiança e se sentir acolhida.* (E6)

A potencialidade do vínculo tem impactos significativos no cuidado da mulher, promovendo esforços conjuntos da equipe na mobilização de estratégias para busca de soluções e atendimento às necessidades de saúde.

*Eu consigo criar um vínculo. Já aconteceu, várias vezes, de ter esse vínculo e conseguir ser eficiente depois nos atendimentos, na resolução do problema e já teve casos até felizes de que a gente conseguiu tirar a mulher daquela situação de drogadição, de rua e conseguiu emprego.* (E6)

### Repercussões do cuidado de mulheres em situação de rua para os enfermeiros

A atenção a mulheres em situação de rua pode sensibilizar os(as) enfermeiros(as) e ocasionar repercussões emocionais.

*Hoje eu entendo que* [...] *pra não pegar o problema pra mim. Porque isso realmente dificulta o nosso trabalho como enfermeiro. Mesmo porque, nós mulheres, nos identificamos com a questão de ser do sexo feminino, então isso realmente acaba doendo um pouco.* (E6)

*Desestrutura um pouco. Porque você vê que é um ser humano, a maioria das vezes são mulheres em idade jovem, fértil e já em uma situação de estar perdida em alguns aspectos de sua vida.* (E8)

*Eu acho que toda a equipe se comove. Não tem como você não se comover.* (E10)

O cuidado do(a) enfermeiro(a) a mulheres em situação de rua promove reflexões sobre a qualidade da assistência, no sentido de identificar o que poderia ser feito além do que já é para essa população, a fim de atender suas necessidades de saúde e promover o cuidado integral.

*Eu fico assim: “meu Deus, o que eu poderia fazer a mais?” Parece que falta alguma coisa. O que eu poderia ajudar mais? Eu sinto que tem uma parte deficiente no trabalho com as mulheres em situação de rua, particularmente.* (E13)

*Não tem como não se sensibilizar e não ficar pensando no que você pode estar fazendo de diferente.* (E12)

*Às vezes, eu fico arrasada, eu fico mal. Eu atendi uma* [usuária] *e eu não sabia que ela vivia em situação de rua e eu não tive um olhar muito acolhedor pra ela naquele momento, tava acontecendo tanta coisa nesse posto* [Unidade Básica de Saúde]*. Depois que acabou* [a consulta de enfermagem] *me falaram que ela vivia em situação de rua e que o principal problema era que ela não tinha comida. Aquele dia eu saí daqui arrasada. Falei: gente, como eu não vi isso?* (E3)

Nesse sentido, os(as) enfermeiros(as) reconhecem a necessidade de formação em serviço e formação inicial dos profissionais para o acolhimento das pessoas em situação de rua e identificação das especificidades na atenção às mulheres.

*A minha principal dificuldade é saber como conversar, o que eu pergunto pra ela, eu acho que tem uma barreira. Elas já ficam meio inseguras e eu acabo ficando também, sobre como vou abordar. Eu não sei a melhor maneira de fazer uma abordagem e conseguir conversar melhor, conhecer mesmo a pessoa, ganhar a confiança. Confesso que eu mesma precisaria de um treinamento, uma capacitação pra isso.* (E12)

*Acho que necessita de mais treinamento, porque, na faculdade em si, não se ensina como acolher uma pessoa em situação de rua, até mesmo porque é muito complexo o atendimento.* (E2)

## DISCUSSÃO

As condições de vulnerabilidade concernentes às mulheres em situação de rua expressam-se em múltiplas necessidades de saúde^(17)^, com a carência de boas condições de vida, inacessibilidade às tecnologias adequadas para manejo do sofrimento, fragilidade nas relações de afeto e vínculo com equipe de saúde e limitada autonomia para levar a vida. Nessa direção, torna-se fundamental fortalecer o uso das tecnologias leves como instrumento terapêutico no cuidado dessas mulheres, cuja humanidade e direitos sociais são violados cotidianamente^(12)^. A promoção da saúde e do bem-estar para todos, especialmente para as populações vulneráveis, é um objetivo fundamental dos ODS. O uso de tecnologias leves como instrumentos terapêuticos está alinhado com a meta de melhorar a saúde mental e o bem-estar das mulheres em situação de rua, além do respeito à dignidade humana e promoção dos direitos de cidadania^(13)^.

As ruas são, entre tantos territórios existenciais, um local onde as existências atuam e se produzem como redes vivas^(26)^. As ruas apresentam códigos específicos e plurais, comportam alegrias, dores, dissabores, desafios e tensões constitutivas entre a produção de vida e morte no cotidiano do andar a vida^(6,26)^. Expressam-se em uma “cultura de rua”, com singularidades e conformações próprias, que usualmente são estranhas aos trabalhadores de saúde, e desafiam os saberes institucionais e as práticas cotidianas dos serviços de saúde ao lidarem com suas existências “incomuns”, para construir uma proposta de cuidado comum, ou seja, compartilhada entre trabalhadores e usuárias^(6)^.

Os encontros entre os trabalhadores de saúde e as pessoas em situação de rua podem ocorrer em uma esfera permeada por tensionamentos, agressividade e desconfiança^(5,12,27,28)^. Muitas vezes, em virtude das condições inóspitas vivenciadas por essas mulheres nas ruas, assim como de experiências negativas nos serviços de saúde, traumas e efeitos de substâncias, elas apresentam comportamentos protetivos, agressivos e tendência de manterem o distanciamento das pessoas^(12,28)^. Nessa direção, o(a) enfermeiro(a) e a equipe de saúde podem encontrar resistência para aproximação e, constantemente, a partir de tensões constitutivas, operam-se negociações entre as usuárias e serviços^(26)^.

Estudos apontam relatos de preconceito, discriminação e desvalorização social de pessoas em situação de rua por parte dos profissionais de saúde^(5,27)^. Pesquisa revela ainda que mulheres em situação de rua possuem histórico como vítimas de violências e que, no cotidiano das ruas, continuam a sofrê-las, perpetradas, inclusive, pelas instituições que deveriam protegê-las, como a polícia^(29)^. Além disso, algumas ações sanitárias de caráter higienista e punitivo estão presentes nos espaços públicos^(30)^.

A resistência, citada pelos(as) enfermeiros(as) por parte das mulheres em situação de rua e das equipes de saúde pode ser interpretada como resultado de um ciclo de produção de violência e de discriminação que precisa ser quebrado, esperando-se que os profissionais de saúde tomem a iniciativa para isso. A escuta com o exercício de não julgamento pode contribuir, mas é preciso o reconhecimento do preconceito e das resistências no cuidado de pessoas em situação de rua^(26)^.

Nesse sentido, o acolhimento foi uma tecnologia leve reconhecida pelo(as) entrevistados(as) para o desenvolvimento do vínculo com as mulheres em situação de rua, que, em um contexto de primeiro contato e longitudinalidade aos serviços de saúde, contribui para a redução das desigualdades^(15)^, condição *sine qua non* para o alcance dos ODS. Nesse caso, a atenção às demandas espontâneas das mulheres em situação de rua, como as queixas ginecológicas, representa uma oportunidade para o cuidado integral, pois permite a identificação e atenção a outras necessidades de saúde em uma perspectiva ampliada e profunda^(9)^. O acolhimento favorece, portanto, a prática clínica do enfermeiro, o desenvolvimento de ações promotoras de saúde, o trabalho em equipe, as relações interprofissionais e entre os usuários e a melhoria no acesso^(15)^. A operacionalização da proposta requer orientação das equipes, para que assumam a responsabilidade de acolher os usuários nas suas demandas, ouvir, negociar e pactuar projetos de cuidado^(20)^.

Muitas mulheres em situação de rua não permanecem em um território geográfico definido, conclamando aos profissionais de saúde à abertura quanto a regras de definição de clientela adstrita, exigência de documentos e de comprovantes de endereço. A complexidade das necessidades de saúde dessa população demanda tecnologias de cuidado que transcendam as rotinas habituais e condutas protocolizadas, guiadas por uma lógica burocrática organizacional que tende a uniformizar as ofertas de cuidado^(6,26)^. Tal lógica também não assegura o atendimento às especificidades e singularidades das mulheres em situação de rua, podendo, inclusive, configurar-se como uma barreira para o acesso aos serviços de saúde^(27)^.

O conceito de território existencial pode ser um eixo norteador para a construção de vínculos entre os(as) enfermeiros(as) e os profissionais de saúde na produção de cuidados para mulheres em situação de rua. A valorização do território para o encontro de interlocutores, com espaço para escuta e fala e com a potencialidade de acolher distintas compreensões de modos de mundo, propicia a abertura de novos caminhos para interferências e intervenções, com repercussões em todos os sujeitos envolvidos, bem como no processo de produção do conhecimento^(6)^.

Denota-se a presença de modos de existências “incomuns” produzidos pelas(os) usuárias(os) que são, muitas vezes, julgados e cerceados pelas equipes de saúde. Por conseguinte, estas ficam aprisionadas a um modo de saber tão preponderante, a ponto de limitarem o reconhecimento de que certas atitudes, comportamentos e expressões são também modos de existência, ainda que se apresentem repletos de tensões e problemas^(26)^. Evidencia-se a frustração por parte dos trabalhadores e equipes de saúde, bem como a sensação de fracasso ao conduzirem ações em cenários adversos, que podem precipitar o abandono ou a tomada de decisões e condutas puramente prescritivas, reduzindo as possibilidades de estabelecimento de vínculo^(6)^.

Ainda, como fator limitante, sublinha-se a influência da cultura do assistencialismo, cujo escopo das ações governamentais é delimitado no âmbito dos problemas imediatos: albergue, alimentação e roupa^(6)^. Trabalhar na perspectiva de garantia de direitos sociais permanece, ainda, um desafio para os governos e suas redes de atenção^(6)^. A implementação de cuidados dignos e respeitosos a pessoas em situação de rua não remete a sentimentos de altruísmo, compaixão ou bondade profissional; trata-se de direitos do usuário, garantidos pela Constituição Federal e pelo SUS^(1,14,23)^.

Um dos desafios apresentados pelos(as) enfermeiros(as) da Atenção Básica para o atendimento de mulheres em situação de rua é justamente a criação do vínculo. Este é considerado uma tecnologia leve, que se refere a uma relação estreita e duradoura entre os profissionais e usuários, com laços afetivos que permitem a continuidade do cuidado^(16)^. A criação de vínculos (a)efetivos entre cada usuária com a equipe e/ou trabalhador de saúde^(17)^ é fundamental para atenção às necessidades de saúde das mulheres em situação de rua.

O modo de operar o trabalho em saúde, em uma perspectiva de simetria no encontro entre diferentes, preza pela valorização da potência singular de cada indivíduo enquanto matéria fundamental para a produção do cuidado^(6)^. Nos encontros permeados por intersubjetividades, torna-se imprescindível conhecer a mulher e tratá-la pelo nome ou apelido de sua preferência, uma vez que a vivência nas ruas transfigura a identidade pessoal e social dos sujeitos, promovendo a sua invisibilidade, exclusão e baixa estima^(12)^. Nessa perspectiva, a valorização da identidade da mulher remete ao encontro e resgate de sua essência humana, dignidade e cidadania^(12,28)^.

O cuidado implica, também, dar voz a essa mulher, valorizá-la, conhecer sua história de vida, razões de encontrar-se em situação de rua, os modos de (sobre)viver e andar a vida nas ruas, bem como compreender suas relações sociais e familiares, as suas singularidades e planos de vida^(12,28)^. Ressalta-se que as usuárias, enquanto redes de existências, produzem-se “em mundos”, “in-mundizam-se”, constituindo expressões éticas existenciais distintas e formas de conduzir, por si, também a produção de cuidado^(26)^. Incorporar o desenho da multiplicidade de vidas aos projetos de abordagem e cuidado das mulheres em situação de rua é tecnologia/saber/prática a ser incorporada pelos profissionais das políticas sociais^(6)^.

A valorização desses encontros, em profundidade, permite a afetação mútua, afetar e ser afetado, ativando estratégias para o viver que apostem na potência da vida, a partir da construção conjunta de soluções para os problemas, em uma via comum para ambas as partes^(6)^. A premissa ética e estética é a de que toda forma de vida tem o direito de expressar-se em sua plenitude e cabe aos serviços de saúde produzir acesso e acolhimento, especialmente a essas mulheres que certamente expressam a desigualdade social, de gênero, racial entre outras em interseccionalidade. Nessa direção, as tecnologias leves se apresentam como práticas de acolhimento, escuta e diálogo, em um campo privilegiado para exercício do autogoverno e reaproximação do fazer em saúde com os projetos de vida das pessoas^(20)^. Garantir que toda forma de vida tenha o direito de expressar-se em sua plenitude está relacionado com a promoção de sociedades pacíficas e inclusivas, conforme previsto no ODS 16. Instituições de saúde eficazes e justas são fundamentais para garantir o respeito aos direitos humanos^(13)^.

Nessa direção, é salutar desenvolver estratégias de educação permanente em saúde para a problematização dos processos de trabalho, analisando o acolhimento das mulheres em situação de rua. Identificou-se, nos discursos dos(as) entrevistados(as), o reconhecimento da necessidade de formação e aperfeiçoamento para o cuidado. Nessa perspectiva, ressalta-se a importância da formação cidadã dos(as) enfermeiros(as), considerando seu papel social, com olhar crítico e reflexivo, conscientes de sua atuação técnica, científica, ética e política^(31)^ no cuidado de mulheres em situação de rua. A construção conjunta de soluções e a importância da formação contínua dos profissionais de saúde ressaltam a necessidade de parcerias eficazes com instituições de ensino e não governamentais. Trata-se de um esforço coletivo para transformar o cuidado e a inclusão social, conforme o ODS 17^(13)^, a fim de promover uma saúde pública inclusiva e equitativa, imprescindível para o desenvolvimento sustentável e a justiça social. Isso exige uma ação coordenada e multissetorial para enfrentar as complexas realidades vividas pelas mulheres em situação de rua.

Os(as) profissionais de enfermagem são agentes fundamentais na transformação de metas globais em ações tangíveis e na promoção de um mundo mais saudável, equitativo e sustentável. Desse modo, quando presentes nos serviços de atenção básica, podem desenvolver práticas profissionais alinhadas e comprometidas com os ODS em seu cotidiano, configurando-se como um pilar essencial na promoção da saúde e no combate às desigualdades. Podem também desempenhar um papel vital na promoção da saúde, na igualdade de gênero, na redução das desigualdades e na construção de sociedades mais justas e pacíficas, ao garantirem o acesso igualitário a cuidados de saúde de qualidade.

### Limitações do estudo

O presente estudo apresentou limitações por restringir-se às entrevistas com os enfermeiros, sem a realização de observação dos atendimentos.

### Contribuições para a Área

Este estudo fomenta reflexões acerca da relevância do uso das tecnologias leves no cuidado de mulheres em situação de rua na Atenção Primária à Saúde, considerando a complexidade de suas necessidades de saúde e como as condições de vida nas ruas impactam no processo saúde-doença e no bem-estar biopsicossocial dessa população. A formação de enfermeiros nos níveis de graduação e pós-graduação, além da formação em serviço, é fundamental para oferecer subsídios para o acolhimento e cuidado integral de uma população invisibilizada e marginalizada, como as mulheres em situação de rua.

## CONSIDERAÇÕES FINAIS

O uso de tecnologias leves de cuidado no cotidiano do trabalho do(a) enfermeiro(a) na Atenção Básica, com a utilização de ferramentas relacionais no cuidado em saúde, tem o potencial de propiciar a criação de vínculos de confiança e diálogo entre as mulheres em situação de rua com os enfermeiros e as equipes. Significa expandir o cuidado para além do espaço “intramuro” da unidade básica de saúde e ir ao encontro dessa mulher, em seu território, nas ruas e reconhecer seus direitos e necessidades. Considera-se que o trabalho na saúde, por ser relacional, depende do trabalho vivo em ato, ou seja, ocorre a partir das relações estabelecidas entre sujeitos trabalhadores e usuários, que acontecem durante o cuidado à saúde. As tecnologias leves podem comandar o trabalho em saúde através da produção de vínculos e acolhimento, aspecto fundamental para o reconhecimento dos direitos das mulheres em situação de rua e vulnerabilidade. Ainda, destaca-se a importância da formação em serviço e da formação inicial dos profissionais para o desenvolvimento de habilidades relacionais, para o reconhecimento do acolhimento e vínculo como tecnologias essenciais para o cuidado às mulheres em situação de rua. Tais elementos são fundamentais para o acolhimento e cuidado integral dessas mulheres, na garantia do direito à saúde e da dignidade humana, elementos fundamentais para a construção de um mundo melhor, de uma sociedade mais justa e inclusiva e para o alcance dos Objetivos do Desenvolvimento Sustentável.

## Data Availability

Os dados da pesquisa estão disponíveis no corpo do artigo.
